# Benthic Nitrogen Loss in the Arabian Sea Off Pakistan

**DOI:** 10.3389/fmicb.2012.00395

**Published:** 2012-11-28

**Authors:** Sarah Sokoll, Moritz Holtappels, Phyllis Lam, Gavin Collins, Michael Schlüter, Gaute Lavik, Marcel M. M. Kuypers

**Affiliations:** ^1^Biogeochemistry Department, Max Planck Institute for Marine MicrobiologyBremen, Germany; ^2^Microbiology, School of Natural Sciences, National University of IrelandGalway, Ireland; ^3^Geosciences, Marine Geochemistry, Alfred Wegener InstituteBremerhaven, Germany

**Keywords:** Arabian Sea, benthic N-loss, anammox, denitrification, qPCR, *nirS*

## Abstract

A pronounced deficit of nitrogen (N) in the oxygen minimum zone (OMZ) of the Arabian Sea suggests the occurrence of heavy N-loss that is commonly attributed to pelagic processes. However, the OMZ water is in direct contact with sediments on three sides of the basin. Contribution from benthic N-loss to the total N-loss in the Arabian Sea remains largely unassessed. In October 2007, we sampled the water column and surface sediments along a transect cross-cutting the Arabian Sea OMZ at the Pakistan continental margin, covering a range of station depths from 360 to 1430 m. Benthic denitrification and anammox rates were determined by using ^15^N-stable isotope pairing experiments. Intact core incubations showed declining rates of total benthic N-loss with water depth from 0.55 to 0.18 mmol N m^−2^ day^−1^. While denitrification rates measured in slurry incubations decreased from 2.73 to 1.46 mmol N m^−2^ day^−1^ with water depth, anammox rates increased from 0.21 to 0.89 mmol N m^−2^ day^−1^. Hence, the contribution from anammox to total benthic N-loss increased from 7% at 360 m to 40% at 1430 m. This trend is further supported by the quantification of *cd_1_*-containing nitrite reductase (*nirS*), the biomarker functional gene encoding for cytochrome *cd_1_*-Nir of microorganisms involved in both N-loss processes. Anammox-like *nirS* genes within the sediments increased in proportion to total *nirS* gene copies with water depth. Moreover, phylogenetic analyses of NirS revealed different communities of both denitrifying and anammox bacteria between shallow and deep stations. Together, rate measurement and *nirS* analyses showed that anammox, determined for the first time in the Arabian Sea sediments, is an important benthic N-loss process at the continental margin off Pakistan, especially in the sediments at deeper water depths. Extrapolation from the measured benthic N-loss to all shelf sediments within the basin suggests that benthic N-loss may be responsible for about half of the overall N-loss in the Arabian Sea.

## Introduction

The Arabian Sea is the semi-enclosed, north-western part of the Indian Ocean. Connected with the Red Sea and the Persian Gulf, it also receives discharge from some of the largest rivers in the world and is fringed by amongst the densest human populations. Although covering only 1% of the ocean surface, the Arabian Sea accounts for ∼5% of the global phytoplankton production, which has characteristic seasonal variability driven by two monsoons each year (Marra and Barber, 2005; Wiggert et al., 2005). Owing to the high seasonal surface production, high respiration in subsurface waters along with slow ventilation produces a pronounced oxygen minimum zone (OMZ) at depths between ∼100 and 1000 m. This OMZ is associated with a high nitrogen deficit (Codispoti et al., 2001; Deutsch et al., 2001) and a strong secondary nitrite maximum found at similar depths, which have been attributed to high pelagic N-loss activities therein (Naqvi, 1994; Naqvi et al., 2006; Ward et al., 2009). Due to its size, the Arabian Sea OMZ is assumed to be one of the biggest pelagic N-sinks, with annual estimated rates varying between ∼30 and 60 Tg N year^−1^ (Bange et al., 2000; Codispoti et al., 2001; Devol et al., 2006).

On the other hand, N-loss processes also occur in marine sediments. In fact, benthic N-loss is believed to contribute ∼50–70% of global oceanic N-loss (Codispoti et al., 2001; Galloway et al., 2004; Gruber, 2004). Because of the geographical configuration of the Arabian Sea, OMZ waters therein impinge on the sediments along the continental margins off the coasts of India, Pakistan as well as Oman. Consequently, any *in situ* N-transformations within the OMZ waters would undoubtedly affect the N-budget of the sediments, and vice versa. Nevertheless, despite the obvious importance of benthic N-loss in the Arabian Sea, benthic N-loss activities have hardly been assessed, and thus estimates of the benthic contribution to the N-deficit and overall N-loss in the Arabian Sea remain poorly constrained. Based on depth-integrated primary production rates (Seitzinger and Giblin, 1996), Bange et al. (2000) estimated that shelf and margin sediments may account for 17% of the N-loss in the Arabian Sea; or up to 26% estimated by Schwartz et al. (2009) from the changes in N_2_:Ar and nitrate consumption rates in Arabian Sea sediments. No direct measurements have been made, however, to distinguish the benthic N-loss pathways, nor have the potential interactions with overlying OMZ waters been much considered.

In general, two processes are known to remove nitrogen from marine systems: the N_2_O and N_2_ production via canonical denitrification ${\text{NO}}_{3}^{-}\to {\text{NO}}_{2}^{-}\to \text{NO}\to {\text{N}}_{2}\text{O}\to {\text{N}}_{2}$ and the N_2_ production by anaerobic ammonium oxidation (anammox, ${\text{NH}}_{4}^{+}+{\text{NO}}_{2}^{-}\to {\text{N}}_{2}$). In marine environments, anammox activities were first detected in sediments (Dalsgaard and Thamdrup, 2002; Thamdrup and Dalsgaard, 2002), and later in the suboxic water columns of the Black Sea (Kuypers et al., 2003) and Golfo Dulce, Costa Rica (Dalsgaard et al., 2003). Since then anammox bacteria have been found in marine habitats ranging from the Arctic sea ice (Rysgaard et al., 2004) to deep sea hydrothermal vents (Byrne et al., 2009). In sediments, anammox has been shown to contribute up to 80% to the N_2_ production (Dalsgaard et al., 2005), but anammox rates measured by ^15^N stable isotope pairing experiments in sediments underlying a major OMZ to our knowledge have never been made before.

The reduction of nitrite to nitric oxide is an essential step in both anammox and denitrification, and is mediated by specific nitrite reductases (Nir; Schalk et al., 2000; Strous et al., 2006; Kartal et al., 2011). In general, two different types of nitrite reductases are known to occur, the copper-(NirK), and the *cd_1_*-containing nitrite reductase (NirS), but organisms harbor either of the reductases. The *nirK* genes are not only present in denitrifiers, but also known to occur in nitrifiers and therefore not suitable for the quantification of denitrifiers. Hence, genes encoding for the (*nirS*) are more commonly used as biomarkers for denitrifiers (Jayakumar et al., 2004; Castro-Gonzalez et al., 2005; Tiquia et al., 2006; Dang et al., 2009) and found to be more abundant in general and in an estuary system (Abell et al., 2010). Meanwhile, anammox bacteria also use a NirS, which is phylogenetically distinct from denitrifier NirS. Thus, *nirS* can be a useful biomarker to distinguish between denitrifiers and anammox bacteria, as evidenced by studies in the Peruvian and Arabian Sea pelagic OMZs (Lam et al., 2009).

In this study, we determined N-loss rates of denitrification and anammox in surface sediments at the continental margin off Pakistan via ^15^N-stable isotope experiments in intact core and slurry incubations. The relative abundances of denitrifying and anammox bacteria in the sediment were quantified based on their respective *nirS* genes and their phylogenies were further evaluated to characterize the benthic microbial communities at various station depths. In order to explore the potential interaction between benthic and OMZ N-loss rates, stations with water depths between 360 and 1430 m were sampled. Accordingly, sediments at one station lay below the OMZ, while the others were within OMZ waters.

## Materials and Methods

### Sampling procedures and chemical analyses

**Figure 1:**
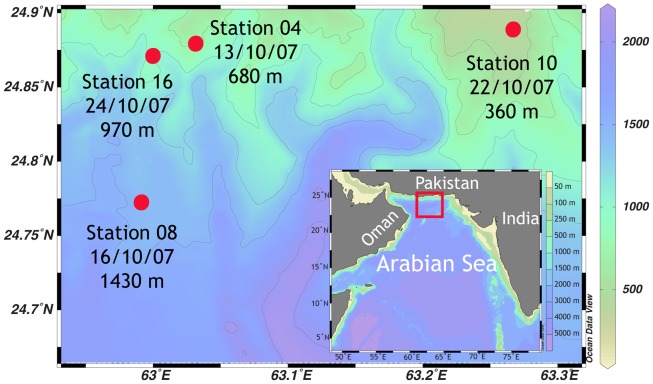
**Sampling area of the *R/V Meteor* cruise M74/2 in October 2007, in the Makran region at the Pakistan continental margin**. Color scale on the right denotes water depth.

Sampling was conducted during the *R/V Meteor* cruise M74/2, on 7th to 28th October 2007, in the Arabian Sea over the Pakistan shelf (Makran region, Figure 1). Four stations ranging from 360 to 1430 m were selected for detailed sampling and sediment incubations. (Please note, that original station names have been shortened for simplicity, from, e.g. GeoB12204 to station 04). Dissolved oxygen and temperature of the water column were measured with a conductivity-temperature-depth (CTD) probe, equipped with an oxygen sensor (Sea Bird Electronics). The oxygen concentration was calibrated against Winkler titration. Water samples were taken with a CTD-rosette. On ship board, concentrations of ammonium and nitrite were measured fluorometrically (Holmes et al., 1999) and photometrically (Grasshoff and Johannsen, 1972), respectively. Additional subsamples were stored at −20°C for later analyses for ammonium, nitrate, nitrite, and phosphate in a shore based laboratory using an autoanalyzer (TRAACS 800, Bran & Luebbe).

Sediment cores were taken with a multicorer (MUC) equipped with eight acrylic liners (10 cm diameter). Subsamples for molecular analyses were taken directly from MUC cores at 2 cm intervals from the surface down to 8 cm. DNA samples were stored at −80°C, shipped on dry ice and kept at −80°C until DNA extraction. Pore water extraction was conducted on board, sediment cores were sliced in a resolution of 0.5 cm (sediment depth 0–1 cm), 1 cm (sediment depth 2–5 cm), and 4 cm (sediment depth 5–0 cm), and pore water was squeezed out of the sediment slices with a pore water press (Schlüter, 1990). Pore water samples for nitrate and nitrite were kept frozen until measurement with a chemiluminescence NO_x_ analyzer (Thermo Environmental Instruments, Inc; Braman and Hendrix, 2002) in a shore based laboratory. For analyses of dissolved iron and sulfide, sediment cores were sampled on board with rhizomes at 1 cm (sediment depth 0–5 cm) and 2 cm intervals (sediment depth 5–30 cm). Subsequently the obtained pore water was analyzed for Fe^2+^ and HS^−^ on board according to Grasshoff et al. (1999) and Cline (1969). Concentrations of organic carbon and nitrogen were determined by combustion/gas chromatography (Carlo Erba NA-1500 CNS analyzer) of dried sediment samples after acidification with 3 mol l^−1^ phosphoric acid in a shore based laboratory.

### Incubation experiments

Benthic denitrification and anammox rates were determined from N_2_ production of ^15^N-labeled slurry and intact core incubations. Rates from slurry incubations were used to calculate the contribution of anammox and denitrification to the total N-loss. Furthermore, volumetric rates from slurry incubations were integrated over the nitrate penetration depth to derive areal N-loss rates. Areal rates were also estimated from intact core incubations according to the revised isotope pairing technique (rIPT) detailed in Risgaard-Petersen et al. (2003).

#### Intact core incubations

Sediment cores (10 cm diameter) were subsampled with 3.6 cm diameter liners and the overlying water was adjusted to a height of 12.5 cm above sediment surface. ${}^{15}{\text{NO}}_{3}^{-}$ (Campro Scientific GmbH) was added to a final concentration of 50 μmol l^−1^ in the overlying water, which was constantly mixed with magnetic stirrers. After pre-incubation for 8–12 h, the cores were sealed with rubber stoppers and incubated without gas phase in the dark at *in situ* temperature (6–16°C). Five time points were taken at ∼0, 2, 6, 10, and 15 h after the cores have been sealed. At each time point, three cores were randomly selected and sacrificed by first removing the rubber stopper and injecting 1 ml of 50% (w/v) zinc chloride to the overlying water to precipitate any free sulfide. Then the first 6 cm of the sediment were mixed with the overlying water. A subsample of the slurry was transferred into 12 ml gas tight sterile glass vials (Exetainer^®^, Labco), poisoned with 100 μl of saturated HgCl_2_ solution to stop biological activity and kept at room temperature in the dark until further processing.

#### Slurry incubations

Vertical distributions of denitrification and anammox rates within the sediment were estimated from slurry incubation experiments in gas tight bags made of plastic-laminated aluminum-foil (Gao et al., 2009). Briefly, MUC sediment cores were sliced in 2 cm intervals between 0 and 8 cm depth. Each slice (volume of ∼160 cm^3^) was transferred into a gas tight bag that was subsequently heat-sealed from all sides. To prepare the slurry, 200 ml of degassed bottom water, taken from the overlying water in the MUC cores, was injected through a gas tight port into the bag. The residual air was removed from the bag and the slurry was thoroughly mixed. After pre-incubating the bags for 2 h, to remove potential air-contamination introduced by the sub-sampling, ^15^N-labeled substrates were injected into the bags and the slurries were again thoroughly mixed. Incubations were performed in the dark at *in situ* temperatures. In Experiment 1, ${}^{15}{\text{NH}}_{4}^{+}$ and ${}^{14}{\text{NO}}_{2}^{-}$ were added to the slurries to final concentrations of 200 and 100 μmol l^−1^, respectively. Furthermore, allylthiourea (ATU) was added to a final concentration of 86 μmol l^−1^ (Ginestet et al., 1998) to inhibit possible bacterial ammonia oxidation. In Experiment 2, ${}^{15}{\text{NO}}_{3}^{-}$ was added to the slurries to a final concentration of 200 μmol l^−1^. For both experiments, a subsample of 6 ml was drawn from the bags immediately after tracer addition, transferred into sterile gas tight glass vials (Exetainer^®^, Labco) and fixed with 100 μl of saturated HgCl_2_ solution. Between five and seven subsamples were drawn from the bags during the subsequent 26–28 h. The exetainers containing the subsamples were kept and shipped upside down in the dark at room temperature.

In the laboratory, a 2 ml helium headspace was introduced into the 12 ml exetainer of the whole core incubations while a headspace of 1 ml was used for the 6 ml exetainer of the slurries. The exetainers were shaken vigorously to allow N_2_ to equilibrate between the headspace and the liquid phase. The N_2_ isotope ratio (^28^N_2_, ^29^N_2_, and ^30^N_2_) of the headspace was determined by gas chromatography-isotopic ratio mass spectrometry (VG Optima, Micromass) by direct injections from the headspace according to Kuypers et al. (2005). Concentrations of ^30^N_2_ and ^29^N_2_ were normalized to ^28^N_2_ and calculated as excess relative to air according to Holtappels et al. (2011). N_2_ production rates were calculated from the ^29^N_2_ and ^30^N_2_ increase over time. Only production with a significant linear slope (*p* < 0.05) over time without delay was used for further calculations.

#### Calculation of N-loss in the sediment slurries

In Experiment 1, the anammox pathway $\left({\text{NH}}_{4}^{+}+{\text{NO}}_{2}^{-}\to {\text{N}}_{2}\right)$ combines either ${}^{14}{\text{NH}}_{4}^{+}\;$or ${}^{15}{\text{NH}}_{4}^{+}$ with ${}^{14}{\text{NO}}_{2}^{-}$ to form ^28^N_2_ and ^29^N_2_. Anammox activity was indicated when the production of ^29^N_2_ (p^29^N_2_) was measured without any production of ^30^N_2_ (p^30^N_2_). The production of ^30^N_2_ was not detected in our samples, only a small amount of ^30^N_2_ production was measured at station 16, depth 2–4 cm. The total N_2_ production via anammox in Experiment 1 [*A*_(Ex1)_] was calculated from:

(1) $$A_{\left(\text{Ex}1\right)}=\frac{{\text{p}}^{29}{\text{N}}_{2}}{F_{{\text{NH}}_{\text{4}}^{+}}}$$

where $F_{{\text{NH}}_{4}^{+}}$ is the labeling percentage of the ^15^N-substrate $\left(F_{{\text{NH}}_{4}^{+}}={\;}^{15}{\text{NH}}_{4}^{+}/({\;}^{14}{\text{NH}}_{\text{4}}^{+}+{\;}^{15}{\text{NH}}_{\text{4}}^{+})\right)$. For Experiment 1, ${\;}^{14}{\text{NH}}_{4}^{+}$ was calculated from the measured ${\;}^{14}{\text{NH}}_{4}^{+}$-concentrations in bottom waters and pore waters and the known addition of ${}^{15}{\text{NH}}_{4}^{+}.$

In Experiment 2, the addition of ${}^{15}{\text{NO}}_{3}^{-}$ to background concentrations of ${}^{14}{\text{NO}}_{3}^{-}$ and ${}^{14}{\text{NH}}_{4}^{+}$ would produce ^28^N_2_ and ^29^N_2_ via anammox and ^28^N_2_, ^29^N_2_, and ^30^N_2_ via denitrification. Thus, the production of ^30^N_2_ (p^30^N_2_) indicates active denitrification. The total N_2_ production by denitrification in Experiment 2 [*D*_(Ex2)_] was calculated according to Thamdrup and Dalsgaard (2002) from p^30^N_2_:

(2) $$D_{\left(\text{Ex}2\right)}=\frac{{\text{p}}^{30}{\text{N}}_{2}}{{\left(F_{{\text{NO}}_{3}^{-}}\right)}^{2}}$$

where $F_{{\text{NO}}_{3}^{-}}$ is the labeling percentage of nitrate $\left(F_{{\text{NO}}_{3}^{-}}{=}^{\;15}{\text{NO}}_{3}^{-}∕{(}^{14}{\text{NO}}_{3}^{-}{+}^{15}{\text{NO}}_{3}^{-})\right).$ In Experiment 2, both, anammox and denitrification produce ^29^N_2_. To calculate anammox from Experiment 2, Eq. 1 is modified to: $A_{\left(\text{Ex}2\right)}=\left({\text{p}}^{29}{\text{N}}_{2}-{\text{p}}^{29}{\text{N}}_{2\left(\text{Den}\right)}\right)∕F_{{\text{NO}}_{\text{3}}^{-}},$ where p^29^N_2(Den)_ is the ^29^N_2_ production via denitrification. With ${\text{p}}^{29}{\text{N}}_{\text{2(Den)}}=2\;{\text{p}}^{30}{\text{N}}_{\text{2}}\left(1-F_{{\text{NO}}_{\text{3}}^{-}}\right)∕F_{{\text{NO}}_{\text{3}}^{-}}$ (Thamdrup and Dalsgaard, 2002), we derive:

(3) $$A_{\left(\text{Ex}2\right)}=\left({\text{p}}^{29}{\text{N}}_{2}-2\frac{\left(1-F_{{\text{NO}}_{3}^{-}}\right)}{F_{{\text{NO}}_{\text{3}}^{-}}}{\text{p}}^{30}{\text{N}}_{\text{2}}\right)\cdot \frac{1}{F_{{\text{NO}}_{\text{3}}^{-}}}$$

Results from slurry incubations indicated the presence of ${\text{NO}}_{3}^{-}$-storing organisms releasing intracellular ${}^{14}{\text{NO}}_{3}^{-}$ in the course of the experiment (for further details, see discussion). An estimate of $F_{{\text{NO}}_{3}^{-}}$ on the basis of measured ${}^{14}{\text{NO}}_{3}^{-}$ bottom water and pore water concentrations was therefore not possible. Instead, we calculated $F_{{\text{NO}}_{3}^{-}}$ from Eq. 3 by inserting the measured p^29^N_2_ and p^30^N_2_ and by assuming *A*_(Ex1)_ = *A*_(Ex2)_. The derived $F_{{\text{NO}}_{3}^{-}}$ value, in the following referred to as ${\;}^{*}F_{{\text{NO}}_{3}^{-}},$ was then used to estimate denitrification according to Eq. 2. For sediments without the release of stored nitrate we expected $F_{{\text{NO}}_{3}^{-}}$ equals ${\;}^{*}F_{{\text{NO}}_{3}^{-}}$, whereas ${\;}^{*}F_{{\text{NO}}_{3}^{-}}<F_{{\text{NO}}_{3}^{-}}$ indicated an additional source of ${}^{14}{\text{NO}}_{3}^{-},$ which was not dissolved initially in the pore water. We denoted the additional nitrate as excess ${}^{14}{\text{NO}}_{3}^{-}$ that was calculated from ${\;}^{*}F_{{\text{NO}}_{3}^{-}}$, $F_{{\text{NO}}_{3}^{-}}$ and the known concentration of ${}^{15}{\text{NO}}_{3}^{-}$ in the slurry:

(4) $$\text{Exces}{\text{s}}^{14}{\text{NO}}_{3}^{-}{=}^{15}{\text{NO}}_{3}^{-}\left(\frac{1}{{}^{*}F_{{\text{NO}}_{3}^{-}}}-\frac{1}{F_{{\text{NO}}_{3}^{-}}}\right)$$

#### Calculation of N-loss in intact sediment cores

From the slurry incubation, the contribution of anammox to the total N-loss was estimated as ra = *A*_(Ex1)_/[*A*_(Ex1)_ + *D*_(Ex2)_]. The total N-loss due to denitrification and anammox was calculated according to Risgaard-Petersen et al. (2003) from ra and the production of ^30^N_2_ and ^29^N_2_ in the core incubations:

$$\begin{array}{llll} \text{N}-\text{loss} & =\text{2}\cdot \frac{\left(1-\text{ra}\right)\;R^{29}-\text{ra}}{2-\text{ra}} & & \\ & \;\cdot \left[{\text{p}}^{\text{29}}{\text{N}}_{\text{2}}+{\text{p}}^{\text{30}}{\text{N}}_{\text{2}}\left(1-\frac{\left(1-\text{ra}\right)\;R^{29}-\text{ra}}{2-\text{ra}}\right)\right] & & \text{(5)} \end{array}$$

where *R*^29^ is the ratio between the ^29^N_2_ and ^30^N_2_ production.

### Detection and phylogenetic analyses of denitrifier and anammox *nirS* genes

The biomarker functional gene *nirS*, encoding the *cd*_1_-containing nitrite reductase, for both denitrifiers and marine anammox bacteria were targeted using qualitative and quantitative analyses. Nucleic acids were extracted from the sediment layers corresponding to those used for rate measurements (0–2, 2–4, 4–6, and 6–8 cm, respectively), by applying the UltraClean^™^ Soil DNA Isolation Kit (MO BIO Labratories, Inc.) according to the manufacturer’s instructions. Triplicate DNA extractions were made for each sample to reduce bias through the extraction procedure and pooled together through purification with the Wizard^®^ Genomic DNA Purification Kit (Promega GmbH). DNA was stored in 10 mM Tris-HCl at −80°C until further analyses. The concentrations of the DNA in the samples were measured spectrophotometrically with a NanoDrop instrument (Thermo Fisher Scientific Inc.).

Denitrifier *nirS* gene fragments were PCR amplified with the primers cd3aF/R3cd (5′-GTSAACGTSAAGGARACSGG-3′ (Michotey et al., 2000)/5′-GASTTCGGRTGSGTCTTGA-3′; Throback et al., 2004). The primers Scnir372F/Scnir845R (5′-TGTAGCCAGCATTGTAGCGT-3′/5′-TCAAGCCAGACCCATTTGCT-3′; Lam et al., 2009) were used to target the specific *nirS* for marine anammox bacteria, so far believed to all fall into the *Candidatus* Scalindua clade. PCR reactions were performed with the Master *Taq* Kit (5 Prime) on a thermal cycler (Eppendorf AG) and were examined with gel electrophoresis on 1% LE agarose gels (Biozym Scientific GmbH).

Subsequently, clone libraries were constructed from PCR amplicons of correct sizes. The PCR products were purified with the QIAquick PCR Purification Kit (QIAGEN) and the cloning reactions were performed with the TOPO TA Cloning^®^ Kit for sequencing (pCR4 vector) with One Shot^®^ TOP10 chemically competent *E. coli* cells (Invitrogen GmbH). Clones were screened for correct inserts by performing PCR with the primers M13F/M13R (5′-GTAAAACGACGGCCAG-3′/5′-CAGGAAACAGCTATGAC-3′), the number of non-nirS sequences was ≤2 per library. PCR products of the correct size were sequenced using the dye terminator sequencing method (Sanger et al., 1977) with the BigDye^®^ Terminator v3.1 Cycle Sequencing Kit (Applied Biosystems, Inc.) and the T7 primer (5′-TAATACGACTCACTATAGGG-3′). Sequencing was performed on an ABI3730 capillary sequencer system (ABI) according to the manufacturer’s protocol. For the primers cd3aF/R3cd, sequence length used for phylogenetic analyses was ∼400 bp, while for primers Scnir372F/Scnir845R sequences had a minimum length of 440 bp.

Sequences were initially processed using BioEdit (Hall, 1999), aligned with ClustalW (Thompson et al., 1994) and screened for NirS encoding genes in the GenBank using the BLAST searches (Altschul et al., 1997). Mothur (Schloss et al., 2009) was used to calculate a similarity cut-off for operational taxonomic units (OTUs) based on nucleic acids of ≥95% and rarefaction curves. The screened sequences were imported into the ARB software package for phylogenetic analyses (Ludwig et al., 2004). Phylogenetic analyses were performed according to the amino acid sequences translated from the obtained sequences, together with some related sequences retrieved from GenBank. Phylogenetic trees were calculated based on the algorithms of maximum likelihood and maximum parsimony. Bootstrapped analyses of 100 resamplings were conducted. The sequences were deposited in GenBank under the accession numbers KC111208 to KC111421.

### Quantitative PCR

Both denitrifier- and *Scalindua*-specific *nirS* genes were further quantified with real-time PCR, using the primers cd3aF/R3cd (Michotey et al., 2000) and Scnir372F/Scnir845R (Lam et al., 2009), which result in amplicons of 425 and 473 bp, respectively. The reactions were performed on an iQ5 cycler (Bio-Rad Laboratories GmbH) with the *Power*SYBR^®^ Green Master Mix (Applied Biosystems Inc.), as previously described (Lam et al., 2009; Jensen et al., 2011). All samples and non-template controls were analyzed as triplicate and the standards were analyzed in every qPCR run. The specificities of PCR amplicons were checked with subsequent melt curve analyses, as well as with 2% agarose gel electrophoresis.

## Results

### Hydro- and geochemistry

**Figure 2:**
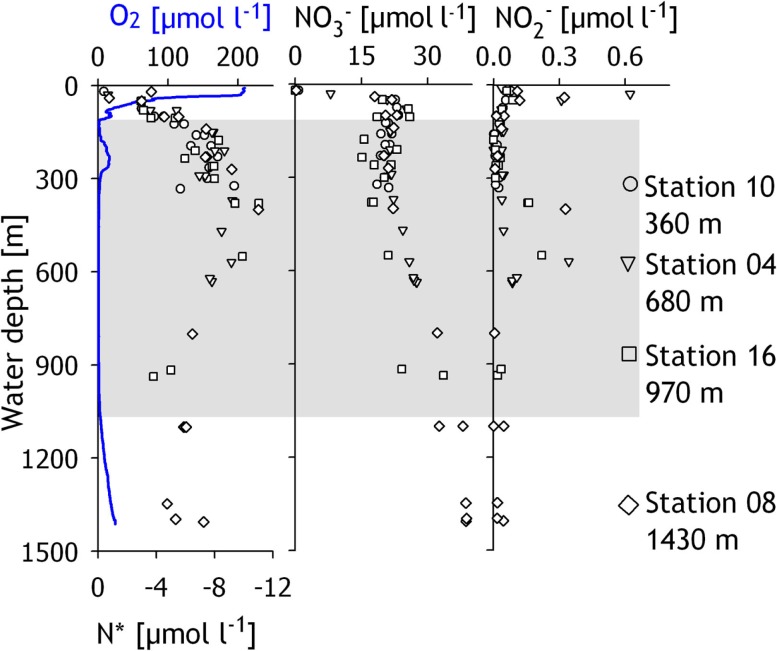
Concentrations of dissolved oxygen, N*, nitrate and nitrite, in the water column of the Pakistan continental margin in the Arabian Sea, with data compiled from all four sampling stations. N* was calculated according to Gruber and Sarmiento (1997). The shaded region depicts the extent of the oxygen minimum zone, while the different symbols denote the data points from different sampled stations.

The compiled oxygen concentration profiles of the four investigated stations revealed an OMZ with a vertical expanse of ∼900 m (Figure 2). Within the oxycline (50–100 m), oxygen concentrations decreased from ∼200 to ∼5 μmol O_2_ l^−1^. From 200 to 300 m, an intrusion of Persian Gulf Water, identified by higher salinity (Shetye et al., 1994), led to increased oxygen concentrations of up to 16 μmol O_2_ l^−1^. At 300 m, oxygen concentrations dropped below the detection limit (∼1 μmol O_2_ l^−1^) and increased again below ∼900 m water depth. Bottom water oxygen concentrations of 23 μmol O_2_ l^−1^ were measured at the deepest station (1430 m), whereas no oxygen was detectable in the bottom water of the three shallower stations. Concentrations of ammonium were low throughout the water column ($<0.1\;\mu \text{mol }{\text{NH}}_{\text{4}}^{+}\;1^{-1}$, data not shown). Nitrite concentrations were close to the detection limit of $0.01\;\mu \text{mol}\;{\text{NO}}_{2}^{-}\;1^{-1}$ but peaked at distinct depths to maximum concentrations of $0.7\;\mu \text{mol}\;{\text{NO}}_{2}^{-}\;1^{-1}$ at 30 m depth and 0.33 μmol l^−1^ between 400 and 600 m (Figure 2). Nitrate was depleted in the surface waters but increased below the oxycline (Figure 2) so that bottom water concentrations increased from $22\;\mu \text{mol}\;{\text{NO}}_{3}^{-}\;1^{-1}$ at the shallowest station to $39\;\mu \text{mol}\;{\text{NO}}_{3}^{-}\;1^{-1}$ at the deepest station. The nitrogen deficit, calculated according to Gruber and Sarmiento (1997) as ${\text{N}}^{*}=\left[{\text{NH}}_{4}^{+}\right]+\left[{\text{NO}}_{2}^{-}\right]+\left[{\text{NO}}_{3}^{-}\right]-16^{*}\left[{\text{PO}}_{4}^{3-}\right]+2.9$, was zero in surface waters (Figure 2), then decreased to −11 μmol N l^−1^ between 300 and 600 m depth and rose slightly to −7 μmol N l^−1^ below 800 m depth.

**Figure 3:**
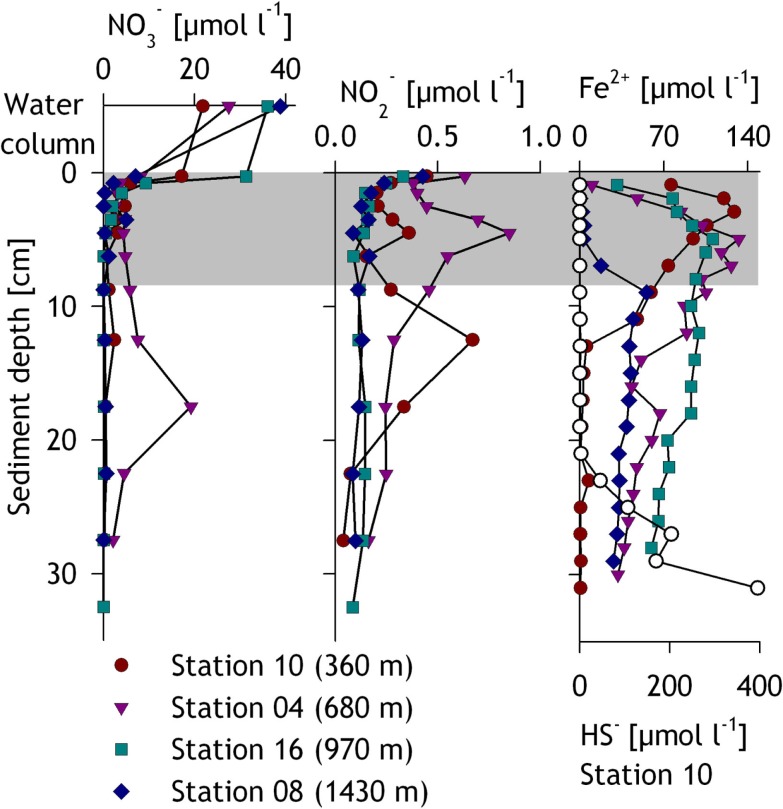
**Pore water profiles for nitrate, nitrite, iron, and sulfide for the investigated stations**. The gray zone indicates the layers sampled for slurry incubations and DNA extraction, while the zone immediately above in the first panel represents the water column or bottom water. Samples for nitrate concentrations in the bottom water were retrieved from the bottom-most CTD sample. Sulfide was only measurable at and thus shown for station 10.

Within the sediments, the pore water was analyzed for the upper ∼30 cm (Figure 3). Nitrate concentrations in the first 0.5 cm of the sediment ranged from $\text{7 to 31}\;\mu \text{mol}\;{\text{NO}}_{3}^{-}\;{\text{1}}^{-\text{1}}$ and dropped sharply to $<3\;\mu \text{mol}\;{\text{NO}}_{3}^{-}\;{\text{1}}^{-\text{1}}$ below. Similar to nitrate, nitrite generally declined within the upper centimeters from $\sim 0.5\;\mu \text{mol}\;{\text{NO}}_{2}^{-}\;{\text{1}}^{-\text{1}}$ at the surface to $0.2\;\mu \text{mol}\;{\text{NO}}_{2}^{-}\;{\text{1}}^{-\text{1}}$ below 2 cm. Significant subsurface maxima of nitrate ($19\;\mu \text{mol}\;{\text{NO}}_{3}^{-}\;{\text{1}}^{-\text{1}}$ at 17.5 cm, station 04) and nitrite (up to $\text{0}\text{.9}\;\mu \text{mol}\;{\text{NO}}_{2}^{-}\;{\text{1}}^{-\text{1}}$, stations 10 and 04) were sometimes found at the shallower stations. Concentrations of dissolved Fe^2+^ increased from 0.2 μmol l^−1^ at the surface to maximum concentrations ranging from 55 to 133 μmol l^−1^ at 5–9 cm depth and decreased within the layers below (Figure 3). Sulfide was detected only at the shallowest station 10 below 23 cm sediment depth where it increased with depth to a maximum of ∼400 μmol HS^−^ l^−1^ at the lowermost sampled layer (31 cm, Figure 3).

Organic carbon and nitrogen contents were measured in the sediment layers corresponding to the slurry incubations (Table 1). Within the OMZ, the organic carbon content (% of dry weight) in the surface sediment layer increased from 1.6% at 360 m to 2.4% at 970 m, but decreased again to 1.7% below the OMZ at 1430 m. Although organic carbon and nitrogen contents decreased within sediment depth at all stations, there was no clear trend for C:N ratios with sediment depth. However, the C:N ratios were slightly enhanced with station depth within the OMZ (C:N = 8–9 at 680 and 970 m), compared to the shallowest and deepest stations (C:N = 7–8).

**Table 1:** **Organic carbon and nitrogen, C:N ratios, N-loss rates, and gene copy numbers of the investigated sediment layers**.

Station	Sediment depth [cm]	Organic carbon [% dry wt]	Organic nitrogen [% dry wt]	C:N [mol:mol]	Excess nitrate [nmol (cm sed)^−3^]	DNA [ng DNA (mg dry sed)^−1^]
10	0–2	1.6	0.26	7.2	95.8	4.92 ± 0.03
	2–4	1.5	0.23	7.8	22.2	3.69 ± 0.23
	4–6	1.5	0.21	8.2	12.3	3.47 ± 0.23
	6–8	1.2	0.21	7.1	n.d.	3.05 ± 0.24
04	0–2	2.2	0.31	8.4	110.6	9.18 ± 0.65
	2–4	2.0	0.28	8.1	23.6	5.04 ± 0.59
	4–6	1.9	0.25	8.8	3.1	6.42 ± 0.84
	6–8	1.8	0.26	8.1	n.d.	3.96 ± 0.33
16	0–2	2.4	0.31	9.0	222.0	5.08 ± 0.14
	2–4	2.1	0.28	8.6	n.d.	5.61 ± 0.49
	4–6	2.0	0.27	8.7	n.d.	4.43 ± 0.43
	6–8	1.3	0.19	8.0	n.d.	3.12 ± 0.41
08	0–2	1.7	0.24	8.3	111.6	6.48 ± 0.41
	2–4	1.6	0.23	7.8	n.d.	5.13 ± 0.21
	4–6	1.4	0.21	7.9	n.d.	5.62 ± 0.40
	6–8	1.2	0.19	7.4	n.d.	2.70 ± 0.08

*n.s., not significant; n.d., not detectable; sed, sediment*.

### Benthic N-loss rates

Benthic N-loss activity was detected in both sediment slurries and intact sediment cores. In the intact core incubations, total benthic N-loss rates increased within the OMZ waters from 0.39 mmol N m^−2^ day^−1^ at 360 m to a maximum of 0.52 mmol N m^−2^ day^−1^ at 680 m (Figure 5A). At the lower boundary of the OMZ, rates decreased to 0.22 mmol N m^−2^ day^−1^ (970 m) and were the lowest at 1430 m (0.18 mmol N m^−2^ day^−1^). The relative contribution of denitrification and anammox to the total N-loss was estimated from slurry incubations. Denitrification rates in intact sediment cores ranged between 0.11 and 0.46 mmol N m^−2^ day^−1^, while anammox rates increased from 0.03 mmol N m^−2^ day^−1^ at the shallowest station to 0.07 mmol N m^−2^ day^−1^ at the deepest station (Figure 5A).

There were strong indications of the release of intracellular ${}^{14}{\text{NO}}_{3}^{-}$ during the slurry incubations. The release of stored ${}^{14}{\text{NO}}_{3}^{-}$ was most apparent in the ${\text{NO}}_{3}^{-}$ measurements in the HgCl_2_-fixed subsamples from the initial time point (*T*_0_). ${\text{NO}}_{3}^{-}$-concentrations at *T*_0_ were significantly above the total sum of ${\text{NO}}_{3}^{-}$ in the bottom water, pore water, and ^15^N-amendment combined, thus indicating an excess of ${\;}^{14}{\text{NO}}_{3}^{-}$ in the slurry. Unfortunately, the true labeling percentage $\left(F_{{\text{NO}}_{3}^{-}}\right)$ during the slurry incubation could not be determined from these subsamples, since any residual intracellular nitrate would have been released after poisoning with HgCl_2_. For this reason, ${\;}^{*}F_{{\text{NO}}_{\text{3}}^{-}}$ was calculated from Eq. 3 (see Materials and Methods) and subsequently the excess concentrations of ${}^{14}{\text{NO}}_{3}^{-}$ were calculated according to Eq. 4. Excess nitrate was calculated for all depths with denitrification rates (Table 1) and generally decreased with sediment depth. Excess nitrate ranged between 222 nmol N (cm^3^ sediment)^−1^ in the surface at station 16 and 3.1 nmol N (cm^3^ sediment)^−1^ in 4–6 cm at station 04.

**Figure 4:**
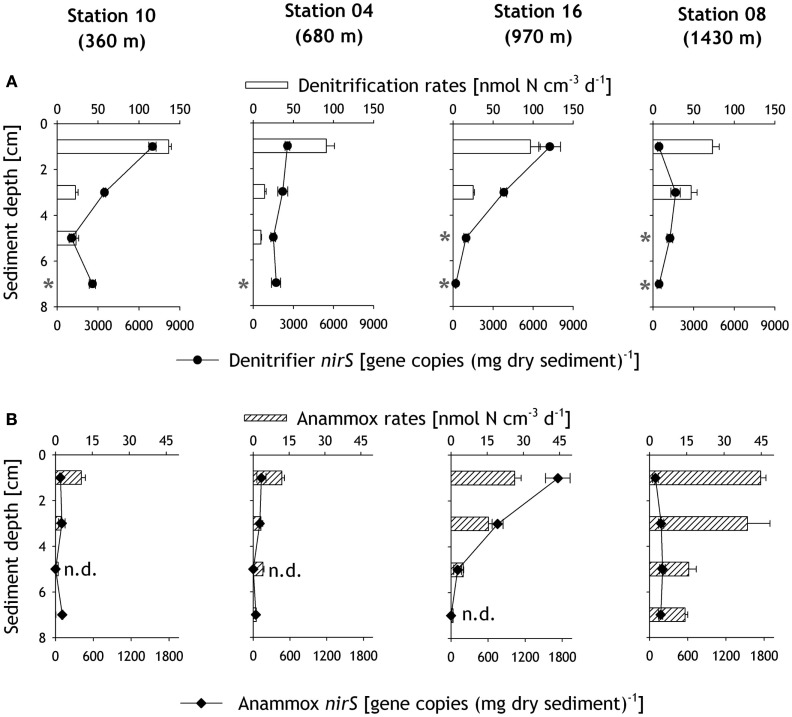
**Denitrification (A) and anammox (B) rates determined from slurry incubation experiments, along with the corresponding gene copy numbers of denitrifier *nirS* (A) and anammox *nirS* (B) quantified by real-time qPCR in the sediments**. The asterisks mark the incubations that showed insignificant ^15^N–N_2_ production. Abbreviations: “n.d.” refers to non-detectable anammox *nirS* gene copy numbers.

**Figure 5:**
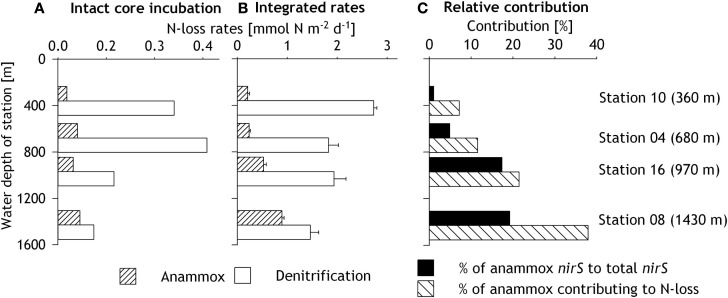
**(A)** Anammox and denitrification rates measured in intact core incubation experiments, using the rIPT method described by Risgaard-Petersen et al. (2003). **(B)** Areal depth-integrated rates of anammox and denitrification measured in slurry incubations for the uppermost 2 cm of the sediment. **(C)** Contribution of anammox to the total benthic N-loss of the slurry incubations and the ratio of anammox *nirS* gene copy numbers to total *nirS* gene copy numbers in the surface layer (0–2 cm) of the sediment.

In slurry incubations, both denitrification and anammox rates generally decreased with increasing sediment depth (Figures 4A,B). Due to insignificant ^29^N_2_ and ^30^N_2_ production, denitrification rates could not be obtained for 6–8 cm at all stations and 4–6 cm at stations 16 and 08. Denitrification rates at the sediment surface (0–2 cm layer) decreased with increasing water depth, from 136 nmol N cm^−3^ day^−1^ at 360 m to 73 nmol N cm^−3^ day^−1^ at 1430 m (Figure 4A). Anammox rates in surface sediments were lower than denitrification rates. However, in contrast to denitrification rates, anammox rates increased with water depth from 10 nmol N cm^−3^ day^−1^ at 360 m to 45 nmol N cm^−3^ day^−1^ at 1430 m (Figure 4B). Anammox and denitrification rates from slurry incubations were integrated down to the nitrate penetration depth of 2 cm (Figure 5B), which represents a rather conservative estimate, given that nitrate was found deeper in the sediment at some stations. Integrated denitrification rates decreased from 2.7 (±0.07) mmol m^−2^ day^−1^ at 360 m to 1.5 (±0.17) mmol m^−2^ day^−1^ at 1430 m. Anammox rates on the other hand increased with water depth from 0.21 (±0.03) mmol m^−2^ day^−1^ at 360 m to 0.89 (±0.04) mmol m^−2^ day^−1^ at the deepest station. As a result, the relative contribution of anammox to total N-loss increased with water depth from 7% at the shallowest station to 38% at the deepest station (Figure 5C).

### Detection of *nirS* genes from denitrifiers and anammox bacteria

**Figure 6:**
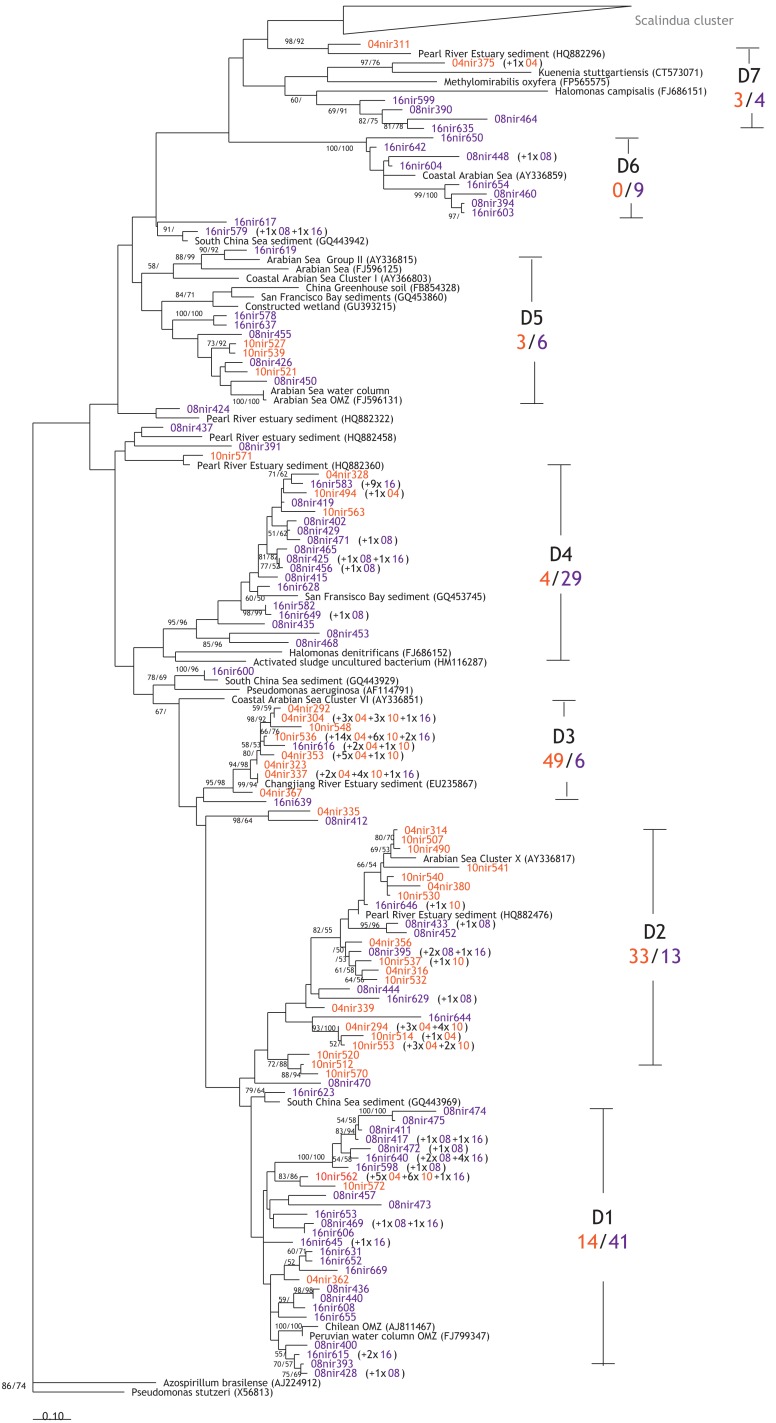
**Phylogenetic reconstruction of the denitrifier NirS based on amino acids sequences translated from gene sequences**. Sequences were retrieved from clone libraries constructed for sediments of all stations based on maximum likelihood and maximum parsimony algorithms. Bootstrapped values of >50% are shown for maximum likelihood/maximum parsimony. Indicated in black are the related reference sequences obtained from GenBank. Labeling of sequences: station = 04, 08, 10, or 16, “nir” = amplicons from primers cd3aF/R3cd, or “sc” = amplicons from primers Scnir372F/Scnir845R, followed by unique sequence number. Numbers in parentheses are the numbers of sequences represented by the same OTU with ≥95% nucleic acids sequence similarity. OTUs from the shallow stations are in orange red, while OTUs from deep stations are in purple. D1–D7 indicate the different clusters identified in this study, while the ratio below gives the ratio of sequences from shallow stations (10 and 04) to the deeper stations (08 and 16).

The presence of microorganisms mediating the denitrification and anammox processes was verified by the detection of their respective biomarker functional genes *nirS*. Altogether, 225 denitrifier *nirS* sequences were obtained, and they formed 114 OTUs that could be grouped into seven clusters (Figure 6; Table A1 in Appendix). The *nirS* sequences from the Pakistan continental margin are diverse, and show clustering pattern that seems to be depth-related: certain clusters are dominated by sequences from the two shallow stations (10 and 04), while others are dominated by sequences from the two deeper stations (16 and 08). The majority of the sequences derived from stations 10 and 04 are found in clusters D2 (33 sequences) and D3 (49 sequences), to which the contributions from the deep stations (08 and 16) are considerably lower (only 13 sequences for D2 and 6 for D3). Meanwhile, clusters D1, D4, D5, and D6 seemed to be dominated by sequences from the deeper stations (08 and 16), with 41, 29, 6, and 9 sequences, respectively. Although the cd3aF–R3cd primer pair amplified predominantly denitrifier *nirS* genes, two sequences (OTU 04nir375) obtained from station 04 (680 m) were found to be more closely affiliated with the freshwater anammox bacterium *Candidatus* “Kuenenia stuttgartiensis” in cluster D7, with a similarity of 73% based on amino acid sequence. It should be noted that cluster D7 also includes cultured species like *Halomonas campisalis* and *Methylomirabilis oxyfera*, which share up to 59 and 69% amino acid sequence similarity, respectively, to the Arabian Sea D7 sequences obtained in this study.

**Figure 7:**
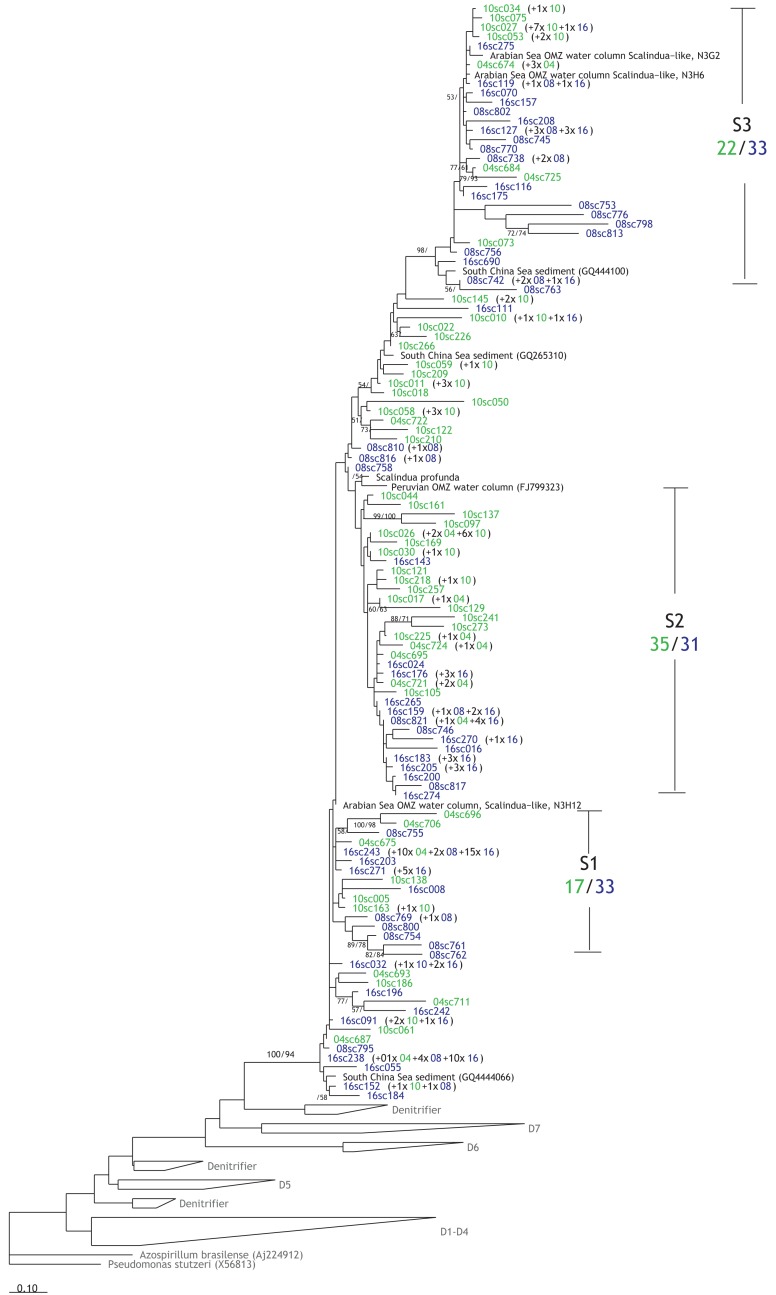
**Phylogenetic reconstruction of the anammox (Scalindua) NirS based on amino acids sequences**. In blue are the OTUs from the deep stations (08 and 16), while the green sequences were obtained from the shallow stations (10 and 04). Please refer to Figure 6 for additional information regarding the sequence labels in tree.

A total of 109 OTUs from 241 anammox *nirS* sequences were retrieved from the Pakistani margin sediments (Figure 7), and they formed three clusters that might also carry some water-depth-related pattern, though not as obvious as for the denitrifier *nirS* sequences. Cluster S1 and S3, closely related to OTUs from the Arabian Sea water column, were dominated by sequences from deep stations (16 and 08) with 33 sequences compared to 17 and 22 sequences from the shallow stations (10 and 04). In contrast, cluster S2 affiliated with OTUs from the Peruvian water column seemed to have similar contributions from both shallow and deep stations.

### Quantification of denitrifier- and anammox-*nirS* genes

Consistent with benthic N-loss rate measurements, the anammox *nirS* genes were generally less abundant than denitrifier *nirS* genes (Figures 4A,B). Both *nirS* gene copy numbers showed a decreasing trend with sediment depth. Amongst all stations, the highest denitrifier *nirS* gene abundance of 7245 ± 813 gene copies (mg dry sediment)^−1^ was detected in the surface sediment layer at station 16 (970 m), whereas the lowest denitrifier *nirS* abundance of 439 (±90) gene copies (mg dry sediment)^−1^ was detected in the uppermost 2 cm at the deepest station 08.

The abundance of anammox *nirS* genes was usually an order of magnitude lower than that of the denitrifier *nirS* (Figure 4B), and was often found to be close to the detection limit. Similar to the denitrifier *nirS* genes, the highest numbers of anammox *nirS* genes were also detected at station 16, ranging from 1728 ± 198 gene copies (mg dry sediment)^−1^ in the surface to undetectable at 6–8 cm. Although the highest rates of anammox were measured in the slurry incubation experiment at station 08, only low gene copy numbers of anammox *nirS*, in the range of 93 ± 44 to 203 ± 44 gene copies (mg dry sediment)^−1^, were detected.

The relative contribution of the anammox *nirS* to the total *nirS* gene copy numbers in the uppermost 2 cm increased with water depth from 1% at 360 m to 19% at station 16 (Figure 5C). These results are consistent with depth-integrated rates, which show an increase of anammox contribution to total N-loss with increasing water depth.

## Discussion

### Benthic N-loss due to denitrification

Consistent with previous benthic N-loss studies from other continental slopes, e.g., the North Atlantic (Trimmer and Nicholls, 2009), denitrification along the Pakistan margin was shown to be the primary N_2_ production process, as measured in slurry incubation experiments and further corroborated by the abundance of the biomarker functional gene *nirS*. Measurements of benthic N-loss rates in the Arabian Sea are rare and so far estimates from direct sediment incubations using ^15^N labeled substrates have not been reported. Schwartz et al. (2009) estimated benthic denitrification rates across the Pakistan continental margin to be 0.40–3.78 mmol N m^−2^ day^−1^. However, these estimates were based on nitrate uptake measurements that would have included the nitrate uptake by nitrate-storing organisms (e.g., sulfur bacteria, foraminifera) as well as the dissimilatory nitrate reduction to ammonium (DNRA). In contrast, N_2_ production rates (determined as the N_2_/Ar ratio) from the same study were lower (0.05–0.13 mmol N m^−2^ day^−1^) than the total N-loss rates we measured with the intact core incubation experiments (0.18–0.52 mmol N m^−2^ day^−1^).

Denitrification rates have been determined for the continental shelf sediments off central Chile, where seasonal hypoxia develops each year (Farías et al., 2004). The measured benthic denitrification rates of 0.6–2.9 mmol N m^−2^ day^−1^ are similar in magnitude to those estimated for the sediments underlying the Peruvian OMZ (0.2–2 mmol N m^−2^ day^−1^) based on modeled pore water fluxes (Bohlen et al., 2011). In comparison, the denitrification rates measured in our intact core incubations for the Pakistan margin (0.11–0.46 mmol N m^−2^ day^−1^) were at the lower end of those estimates for the Chilean and Peruvian sediments, while the integrated rates based on slurry incubations (1.46–2.73 mmol N m^−2^ day^−1^) lay within the upper range. The actual *in situ* N-loss rates on the Pakistan margin are likely somewhere between these two sets of obtained rates – as the amended substrates in the slurry incubations could have stimulated additional N-loss activity, while intact cores might have underestimated N-loss activity due to insufficient diffusion of the ^15^N-labeled substrates into deeper sediment layers. Moreover, intact core incubations could not account for any potential denitrification by nitrate-storing organisms (e.g., foraminifera) as would be discussed below. Therefore, rates derived from slurry incubations may be closer to reality than those from intact core incubations.

Several lines of observations collectively indicate the likely presence of nitrate-storing organisms in the sediments of the Pakistani margin. Firstly, high production of ^29^N_2_ relative to ^30^N_2_ was measured in the ${}^{15}{\text{NO}}_{3}^{-}$ incubations, which did not agree with the calculated labeling percentage and the measured anammox rates. Secondly, nitrate concentrations in the *T*_0_ subsamples of the slurry incubations exceeded the sum of bottom water, pore water and ^15^N-nitrate. Thirdly, subsurface maxima of pore water nitrate/nitrite, similar to those previously observed at the Pakistani margin (Woulds et al., 2009), were found during this study. These various findings combined suggest that intracellular ${\text{NO}}_{\text{x}}^{-}$ had been released during the pore water squeezing and during the mixing of sediment slurries.

Nitrate-storing sulfur bacteria, such as *Thioploca* spp. and *Beggiotoa* spp., have been associated with high pore water nitrate concentrations (Fossing et al., 1995). However, despite the lack of detailed microscopic or molecular analyses to confirm their absence, these mat-forming sulfur bacteria were not visible to naked eyes in the collected samples. Besides, sulfide was only detectable at the shallowest station (station 10) and only below 23 cm, while there were high concentrations of Fe^2+^ at all other stations that indicated the absence of free sulfide. Given such low availability (or lack) of electron donor for their energy production, it was thus unlikely for these sulfur bacteria to thrive in the sediments examined. On the other hand, nitrate storage of up to 80% of the total benthic nitrate pool has been described for foraminifera in sediments (Risgaard-Petersen et al., 2006; Glud et al., 2009), including the Peruvian OMZ (Piña-Ochoa et al., 2010). Indeed, living foraminifera had been found particularly in the first cm of sediments underlying the OMZ at the Pakistan margin (Schumacher et al., 2007), which agrees well with the enhanced excess nitrate concentrations calculated for the uppermost sediment layer in our samples. The mean excess nitrate concentration in our study was ∼135 nmol (cm^3^ sediment)^−1^, equivalent to twice as much as that reported in the anoxic zone of Gullmar Fjord, Sweden [∼60 nmol (cm^3^ sediment)^−1^; Risgaard-Petersen et al., 2006].

Denitrification from the stored ${\text{NO}}_{3}^{-}$ by foraminifera would lead to false denitrification estimates if the intracellular labeling percentage $\left(F_{{\text{NO}}_{3}^{-}}\right)$ was not known. However, the increased ${\text{NO}}_{3}^{-}$ concentrations in the slurry subsamples at *T*_0_ suggest that the stored ${\;}^{14}{\text{NO}}_{3}^{-}$ was released into the pore water when the slurry was mixed at the start of the experiment. Thus, a subsequent uptake of ${\text{NO}}_{3}^{-}$ from the pore water would lead to an intracellular $F_{{\text{NO}}_{3}^{-}}$ that is close to the pore water $F_{{\text{NO}}_{3}^{-}}$. Furthermore, the linear increase of ^29^N_2_ and ^30^N_2_ with time indicates that either intracellular $F_{{\text{NO}}_{3}^{-}}$ did not change over time or that the N_2_ production by foraminifera was minor, as was also observed in other regions (Glud et al., 2009). Nonetheless, nitrate-storing foraminifera would potentially lead to an underestimation of N-loss by intact core incubations, since the unlabeled intracellular nitrate was not accounted for. In order to fully explain the source of excess nitrate observed, additional sample collection and analyses, including some shipboard microscopic examination of live cells, would be necessary to especially target the nitrate-storing sulfur bacteria and foraminifera at the point of sampling. These were unfortunately unavailable in our current study and should be further investigated.

The dominance of denitrification in benthic N-loss in the Pakistan margin sediments is strongly supported by the high abundance of denitrifier *nirS* genes. Moreover, the gene copy numbers generally followed similar decreasing trends as the rates measured in slurry incubation within the sediments (Figure 4). Exceptions were noted particularly in the topmost layer(s) at the deepest station (station 8), and these could potentially be due to nucleic acid extraction efficiency or biases, and/or the presence of PCR inhibitors. In addition, the primers used only target *nirS*, while any occurrence of the *nirK* genes would not have been accounted for. Although there are also primers designed for *nirK*, those currently available may also target those of nitrifiers. Consequently, quantification of *nirK* in addition to that of *nirS* would likely overestimate denitrifier abundance instead. Future refinement of primer designs, or the assessment of multiple biomarker genes in parallel, may help shed light on the true quantitative distribution of denitrifiers in the environment. Compared to previous studies in various sediments, most of which also focused on denitrifier *nirS* and found gene copy numbers ranging from ca. 0.6 × 10^3^ copies (mg sediment)^−1^ at the mouth of the Colne estuary (Smith et al., 2007) to 27.2 × 10^3^ copies (mg sediment)^−1^ at the mouth of the Rhône River (Michotey et al., 2000), denitrifier *nirS* abundance at the Pakistan margin [0.2–6.9 × 10^3^ copies (mg sediment)^−1^] lay within the same range.

**Figure A1:**
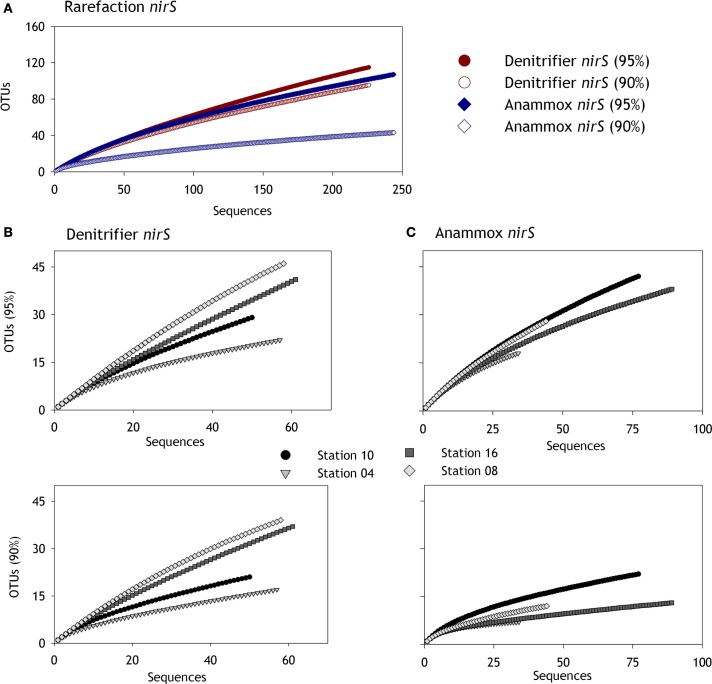
**Rarefaction curves for denitrifier and anammox (Scalindua) nirS sequences based on 95% and 90% nucleotide sequences cut offs**. **(A)** Shows rarefaction curves over all sampled stations, **(B)** shows the denitrifier *nirS* rarefaction curves separately for each sampled station, and **(C)** the corresponding curves for anammox (Scalindua) *nirS*.

In agreement with studies addressing *nirS* genes in the water column of the Arabian Sea (Jayakumar et al., 2004; Bulow et al., 2008), the denitrifier *nirS* community seems to be very diverse (Chao1 richness estimate = 327). However, diversity seems to vary amongst the stations (Chao1 richness estimates of 48–239 were calculated), though the rarefaction analyses indicate that the sequences obtained from the two deeper stations may not be sufficient to represent the full denitrifier diversity therein (Figures A1A,B in Appendix). Phylogenetic analyses revealed some apparent differences in the shallow versus deep denitrifying communities, with certain clusters dominated by sequences from shallow stations, while others by sequences from the deeper stations (Figure 5). As suggested in other studies (Liu et al., 2003; Dang et al., 2009), such a clustering pattern could result from the adaptation of specific denitrifying communities to different environmental conditions that vary with water depth, such as oxygen, carbon, and nitrate availabilities.

It is particularly interesting to find an OTU amplified with the primers targeting denitrifier *nirS* genes, to be related to the *Ca*. “K. stuttgartiensis” (73% similarity, Figure 6). *Ca*. “K. stuttgartiensis” is an anammox bacterium known to occur in freshwater (Jetten et al., 2003), though capable of adapting to higher salinity (Kartal et al., 2006), it has never been found in marine environments thus far. In the same cluster (D7), between the Scalindua cluster and a cluster (D6) affiliated with a sequence from the Arabian Sea water column (Jayakumar et al., 2004), sequences from the deep stations are most closely affiliated with the halophilic bacteria *H. campisalis* (Mormile et al., 1999) and *M. oxyfera*, a freshwater methanotroph that denitrifies via an alternative pathway (Ettwig et al., 2010). The interesting NirS phylogeny of the cluster D7 may indicate that these organisms were no ordinary denitrifiers, yet their exact metabolic pathways remain to be determined. Recent studies from a hydrothermal vent system (Byrne et al., 2009) and an estuary (Dang et al., 2010) report the presence of anammox bacteria, other than *Candidatus* “Scalindua.” These results together with the finding of the OTU related to *Ca*. “K. stuttgartiensis” in this study may hint toward a different type of anammox bacteria, although the abundance seems to be very low. Further studies need to be conducted to verify the occurrence of anammox bacteria, other than *Candidatus* “Scalindua” in the marine environment.

### Benthic N-loss via anammox

This study provides the first direct measurement of anammox activity in the sediments of the Arabian Sea, or any OMZs. The very recent study by Bohlen et al. (2011) in the Peruvian OMZ estimated benthic anammox rates based on modeled pore water fluxes of up to 0.43 mmol N m^−1^ day^−1^ for an anoxic station at 376 m, with lower rates at deeper as well as shallower stations. In general, anammox rates according to intact core incubations at the Pakistan continental margin are much lower (0.003–0.007 mmol N m^−1^ day^−1^) than the estimates from the Peruvian OMZ. The integrated anammox rates based on slurry incubations, on the other hand, are comparable (0.21 mmol N m^−1^ day^−1^) on the Pakistan margin at a similar water depth (360 m) and reached as high as 0.89 mmol N m^−1^ day^−1^ at the deepest sampled station (1430 m). In congruence with the rate measurements, anammox Scalindua-like *nirS* genes could be detected at all stations and are in lower abundance than the denitrifier *nirS* genes. The anammox *nirS* gene abundance [undetectable to 1.7 × 10^3^ copies (mg sediment)^−1^] detected at the Pakistan margin were an order of magnitude lower than those detected in deep sea sediments of South China Sea [up to 44.1(±3.3) × 10^3^ copies (mg sediment)^−1^; Li et al., 2011] in which the same primers were used as in the current study.

Because the *nirS* gene is present as a single copy in anammox bacteria, according to the sequenced genomes of both the freshwater *Ca*. “K. stuttgartiensis” (Strous et al., 2006) and marine *Candidatus* “Scalindua profunda” (van de Vossenberg et al., 2008), potential cell specific activity may be calculated from the anammox rates measured in slurry incubations and the anammox *nirS* gene copies quantified. Taking station 16 that lay within the OMZ as an example, cell specific anammox rates were calculated to be 10–24 fmol N cell^−1^day^−1^, which was highly similar to those estimated for the Arabian Sea OMZ waters (1.6–25 fmol N day^−1^ cell^−1^) over the Omani Shelf (Jensen et al., 2011). However, likely lower DNA extraction efficiency in sediments has probably led to underestimated anammox *nirS* gene copy numbers particularly for the deepest station, which in turn would result in overestimated cell specific rates, and so are not presented here. In addition, a recent study reported the occurrence of *nirK* instead of *nirS* gene in a freshwater anammox bacterium from a bioreactor (Hira et al., 2012). Although *nirK*-containing anammox bacteria have not been found in the marine environment to date, such possibility cannot be eliminated and the quantification of *nirS* genes alone might have underestimated the anammox bacterial abundance. In future studies, the recently discovered gene *hzsA*, encoding hydrazine synthase (Harhangi et al., 2012), might be a reasonable alternative or additional biomarker gene for the quantification of anammox bacteria, since it is also present as a single copy in the genomes analyzed until now.

According to the phylogenetic reconstruction of Scalindua NirS (Figure 6), three different clusters could be identified and the diversity of the community (Chao1 richness estimate = 275), though lower than the diversity of the denitrifier NirS (Figures A1A,C in Appendix), seems to be higher compared to those found in the water column OMZ of the Arabian Sea (Chao1 richness estimate = 8; Jensen et al., 2011) and Peru (Chao1 richness estimate = 43; Lam et al., 2009). The higher diversity could have been caused by more distinct segregation of the organisms in the sediments compared to the water column. Similar to the denitrifier NirS tree, sequences from the deep stations appeared to predominate in two clusters, presumably due to their different adaptations to environmental conditions as mentioned earlier for the denitrifiers.

### Anammox contribution increased with water depth

In agreement with other studies (Engström et al., 2009; Trimmer and Nicholls, 2009; Bohlen et al., 2011), we found an increasing contribution of anammox to the total benthic N-loss with increasing water depth. At a water depth of 1430 m, the contribution of anammox was the highest (38%) and similar to the mean anammox contribution of 37% measured by Glud et al. (2009) at comparable water depths (1450 m) in a basin with low oxygen concentrations (∼60 μmol O_2_ l^−2^) off Japan (Sagami Bay). Even at the Washington margin with water depths >2700 m, the contribution of anammox to total N-loss was found to be 40% on average (Engström et al., 2009). These studies, all based on ^15^N incubation experiments, suggest a consistent contribution of ∼40% of anammox to the benthic N-loss at sites with water depths >1400 m in different regions across global oceans. Earlier studies, as summarized by Trimmer and Engström (2011), observed a decrease in both denitrification and anammox rates with increasing water depth, such that the overall increase in anammox contribution to total N-loss with water depth was attributed to less decrease in anammox activity relative to denitrification. In contrast, this study shows an increase of potential anammox activity in the slurry incubation experiments from 0.21 to 0.89 mmol N m^−2^ day^−1^ with station depth, while denitrification rates decreased from 2.7 to 1.5 mmol N m^−2^ day^−1^. This trend was further corroborated by the relative increase in anammox *nirS* gene copy abundance with the water depth (Figure 7).

Although anammox rates and cell abundance increase with water depth, it is unlikely that water depth or rather pressure itself is a direct regulating factor for the anammox contribution, since bacterial communities and denitrifiers in particular are able to cope with high pressure very well (Tamegai et al., 1997). More likely than pressure are factors that correlate with depth, such as temperature, organic carbon content, and nitrate concentration. Trimmer and Nicholls (2009) attributed the increase of anammox contribution to total N-loss, amongst other factors, to the bottom water temperature. Experiments with different incubation temperatures suggested, that anammox might be more compatible with lower temperatures (Dalsgaard and Thamdrup, 2002). This could also be the case here as the measured bottom water temperature at the Pakistan margin decreased with the water depth from 15.7°C at the shallowest station to 6.1°C at the deep station. On the other hand, it is generally believed that temperature and metabolic rates correlate (Gillooly et al., 2001, 2002; Savage et al., 2004) such that temperature is unlikely the responsible factor for the increase in anammox rates with depth at the Pakistan margin.

Organic carbon concentrations usually decrease with water depth and therefore it is hypothesized in some studies (Nicholls and Trimmer, 2009) that a decrease in benthic carbon content favors the chemolithoautotrophic anammox process. In the meantime, denitrifiers seem to proliferate particularly in reactive sediments where the lability as well as content of organic matter are higher (Engström et al., 2005), due to their possibly stronger competition for nitrite as electron acceptor when the electron donors (i.e., organic matter) are abundant. However, at the Pakistan margin, benthic organic carbon content of surface sediments did not show a decreasing trend with water depth, but increased within the core OMZ. It has been suggested that downslope redistribution of shelf sediments and increased preservation of organic carbon under anoxic conditions have caused the high organic carbon content in the core OMZ (Schott et al., 1970). Indeed, the highest organic carbon content was found along with the highest C:N ratio at the bottom of the OMZ (station 16), which hints toward the assumption that the organic matter is more refractory. Unlike the dependence of heterotrophic denitrifiers on the availability of labile organic carbon, anammox bacteria can fix their own organic carbon and therefore likely have an advantage at the deeper stations, where the supply of organic carbon from the surface is lower due to probably reduced primary production with distance to the coast and/or greater extent remineralization in the water column reaching those depths.

Anammox activity depends on sufficient supplies of ${\text{NO}}_{\text{x}}^{-}$ (Dalsgaard and Thamdrup, 2002), which acts as the electron acceptor for the anammox reaction. The highest anammox rates were measured at the deepest station, where nitrate concentration was almost twice as high (∼39 μmol l^−1^) as at the shallow station in the upper OMZ (∼22 μmol l^−1^). Moreover, oxygen was present which could have stimulated nitrification and thus could enhance the availability of ${\text{NO}}_{\text{x}}^{-}$ in the sediments. The high nitrate concentrations and to a lesser extent the more refractory organic carbon at the deeper stations could have led to incomplete denitrification (i.e., nitrate reduction to nitrite) and an overall increased availability of ${\text{NO}}_{\text{x}}^{-}$ for anammox (Dalsgaard et al., 2005). This would be particularly important for deeper sediment layers, where ${\text{NO}}_{\text{x}}^{-}$ availability is usually low. This postulation would be in good agreement with the high rates measured in deeper layers at station 08 (Figure 4B), the deepest station with the highest nitrate concentration and oxic overlying bottom water.

### Contribution of benthic N-loss to the N-deficit in the Arabian Sea

In general, the water column of the central Arabian Sea is believed to be an important sink for fixed nitrogen in global oceans as indicated by a prominent N-deficit (Naqvi, 1994; Naqvi et al., 2006; Ward et al., 2009). Recent studies on the water column N-loss in the Arabian Sea OMZ could not agree on the dominant pathway, denitrification or anammox, responsible for the N-loss therein, and much variability has been found in the measured rates (Ward et al., 2009; Jensen et al., 2011; Lam et al., 2011). Ward et al. (2009) measured pelagic denitrification of up 25.4 nmol N_2_ l^−1^ day^−1^ in the central Arabian Sea. In contrast, pelagic N-loss rates measured during the cruise for this study at the same stations on the Pakistan margin (data not shown here) as well as in the central Arabian Sea (Jensen et al., 2011) immediately before this study were very low (0–0.04 nmol N l^−1^ day^−1^). These direct rate measurements together may suggest that the Arabian Sea harbors distinct regions of seasonally high N-loss (Lam et al., 2011), rather than being an area of uniformly and persistently high N-loss activity throughout the year. While the water column seems to be subject to seasonal variations in N-loss due to the supply of substrates from the surface and removal by sinking particles, benthic N-loss is likely less seasonally dependant, since organic carbon concentrations integrate over a longer period of time. Hence, consistently high benthic N-loss may have contributed significantly to the N-deficit signals in the water column where the OMZ water impinges on the Pakistani margin.

Naqvi et al. (2006) calculated that an area of 1.15 m × 10^12^ m of seafloor in the Arabian Sea is affected by oxygen concentrations of <22 μmol O_2_ l^−1^. Since we measured N-loss at four stations across the OMZ with bottom water O_2_ concentrations of 0–23 μmol l^−1^, an extrapolation of average fluxes to the area estimated by Naqvi et al. appears reasonable. The mean rates measured in the slurry incubations in this study would result in an annual N removal as high as 14.7 Tg N year^−1^ (range between 12.3 and 17.0 Tg N year^−1^). Similar rates via denitrification of 1.1–10.5 Tg N year^−1^ were estimated for the continental shelves of the Arabian Sea by Schwartz et al. (2009). Based on primary production rates, Bange et al. (2000) estimated the N-loss from shelf sediments (0–200 m) to be 6.8 Tg N year^−1^ and as much as 33 Tg N year^−1^ were attributed to pelagic denitrification. Accordingly, shelf sediments would account for only 17% to the total N-loss in the Arabian Sea. Nonetheless, these estimates did not include sediments at water depths deeper than 200 m, which also contribute to the N-loss in the Arabian Sea. Therefore, sediments likely contribute more to the total N-loss in the Arabian Sea than previously assumed.

Furthermore, N-loss rates measured in the central Arabian Sea of 0.3–0.6 mmol N m^−2^ day^−1^ (Jensen et al., 2011) are comparable to benthic N-loss rates measured in this study. An extrapolation of these rates to the area of the Arabian Sea to the north of 6°N (4.93 × 10^12^ m^2^; Bange et al., 2000) would result in an annual pelagic N-loss of 7.6–15 Tg N year^−1^, which is similar to a recently published estimate for pelagic N-loss in the Arabian Sea of 12–16 Tg N year^−1^ (DeVries et al., 2012). Compared to the mean benthic N-loss calculated from our data (14.7 Tg N year^−1^) with only the shelf sediments included, water column and the sediments might contribute more or less equally to the N-loss in the Arabian Sea.

## Conclusion

Benthic N-loss due to anammox increased with water depth on the Pakistan margin and the contribution of anammox to total N-loss seemed to co-vary with temperature and nitrate concentrations in the bottom water. Compared to shallow sediments, anammox bacteria seem to be more successful in deeper sediments, as anammox accounted for almost 40% to the total benthic N-loss at 1430 m water depth. The shift from a denitrifier-dominated heterotrophic system in shallow sediments, to a system in which the autotrophic anammox process plays a more important role in sediments at deeper water depths, could also be coupled to the availability of labile organic carbon. Owing to their chemolithoautotrophic lifestyle, anammox bacteria could have a competitive advantage over denitrifiers in deeper sediments due to their lesser dependence on the often seasonally fluctuating primary production in surface waters for sources of electron donor and carbon. Extrapolation from our data suggests that benthic N-loss could account for up to half of the total N-loss in the Arabian Sea as a whole, and may thus have contributed to the N-deficits in the water column, though further investigations during different seasons are necessary to fully evaluate the role of sediments in the annual marine N-loss. Since human populations and anthropogenic atmospheric N deposition (Duce et al., 2008) have been increasing in the Arabian Sea, primary production therein would likely be enhanced further in the near future, possibly resulting in higher oxygen consumption and thus an expansion of the OMZ. What additional positive and negative feedbacks may ensue, and how the overall nitrogen as well as the intimately linked carbon cycles might respond in this key region of global biogeochemical cycling, cannot be fully evaluated without taking the interacting benthic and pelagic fluxes into due consideration.

## Conflict of Interest Statement

The authors declare that the research was conducted in the absence of any commercial or financial relationships that could be construed as a potential conflict of interest.

## Appendix

**Table A1:** **Range of similarities (%) between the different clusters identified in the NirS phylogenetic trees, based on amino acid sequences**.

	Denitrifier or *Kuenenia*-like									*Scalindua*-like			
	D1	D2	D3	D4	D5	D6	D7	*Kuenenia stuttgartiensis*	*Methylomirabilis oxyfera*	S1	S2	S3	*Scalindua profunda*
D1													
D2	82–54												
D3	82–60	76–56											
D4	70–47	71–53	70–54										
D5	74–51	70–55	70–56	72–55									
D6	59–38	60–47	58–44	62–47	63–48								
D7	58–38	58–43	59–41	58–46	61–45	66–48							
*Kuenenia stuttgartiensis*	61–48	60–53	61–54	62–55	62–53	60–51	73–38						
*Methylomirabilis oxyfera*	58–42	58–49	55–45	59–52	60–54	62–47	69–42	56					
S1	62–39	61–42	57–39	63–44	66–47	66–46	60–44	61–51	64–56				
S2	61–41	62–44	57–40	65–43	68–48	65–49	63–44	61–50	66–54	94–57			
S3	66–38	62–37	59–36	65–38	67–44	65–41	63–42	59–44	64–49	85–52	84–50		
*Scalindua profunda*	60–49	61–52	57–47	64–54	65–57	64–56	62–45	51	60	94–77	95–77	82–64	
